# Rapidly Improving High Light and High Temperature Tolerances of Cyanobacterial Cell Factories Through the Convenient Introduction of an AtpA-C252F Mutation

**DOI:** 10.3389/fmicb.2021.647164

**Published:** 2021-04-08

**Authors:** Shanshan Zhang, Sini Zheng, Jiahui Sun, Xuexia Zeng, Yangkai Duan, Guodong Luan, Xuefeng Lu

**Affiliations:** ^1^Key Laboratory of Biofuels, Qingdao Institute of Bioenergy and Bioprocess Technology, Chinese Academy of Sciences, Qingdao, China; ^2^Shandong Provincial Key Laboratory of Synthetic Biology, Qingdao Institute of Bioenergy and Bioprocess Technology, Chinese Academy of Sciences, Qingdao, China; ^3^College of Life Science, University of Chinese Academy of Sciences, Beijing, China; ^4^College of Life Science and Technology, Central South University of Forestry and Technology, Changsha, China; ^5^Dalian National Laboratory for Clean Energy, Dalian, China; ^6^Laboratory for Marine Biology and Biotechnology, Qingdao National Laboratory for Marine Science and Technology, Qingdao, China

**Keywords:** cyanobacteria, *Synechococcus elongatus* PCC 7942, ATP synthase, sucrose, high temperature and high light tolerances

## Abstract

Photosynthetic biomanufacturing is a promising route for green production of biofuels and biochemicals utilizing carbon dioxide and solar energy. Cyanobacteria are important microbial platforms for constructing photosynthetic cell factories. Toward scaled outdoor cultivations in the future, high light and high temperature tolerances of cyanobacterial chassis strains and cell factories would be determinant properties to be optimized. We proposed a convenient strategy for rapidly improving high light and high temperature tolerances of an important cyanobacterial chassis *Synechococcus elongatus* PCC 7942 and the derived cell factories. Through introduction and isolation of an AtpA-C252F mutation, PCC 7942 mutants with improved high light and high temperature tolerances could be obtained in only 4 days with an antibiotics-free mode. Adopting this strategy, cellular robustness and sucrose synthesizing capacities of a PCC 7942 cell factory were successfully improved.

## Introduction

With resource shortages and environmental pollution issues becoming increasingly prominent, photosynthetic biomanufacturing provides important options for the green and sustainable production of biofuels and biochemicals (Melis, 2009; Lu, 2010). Cyanobacteria are potential photosynthetic platforms and have been successfully engineered for production of multiple natural and non-natural products (Oliver and Atsumi, 2014). To put cyanobacterial photosynthetic biomanufacturing technology into practice, high light and high temperature tolerances would be important properties of the cyanobacterial cell factories, aiming to facilitate stable growth and production under stressful conditions during scaled outdoor cultivations (Luan and Lu, 2018). To improve high light and high temperature tolerances of cyanobacterial cells, complex genetic engineering strategies have been explored and adopted to modify or to update the native stress-response protective systems or the photosystems. For example, overexpression of specific heat shock proteins improved high temperature tolerance of cyanobacterial cells (Chaurasia and Apte, 2009; Su et al., 2017), and reduction of light harvesting antenna led to improved tolerances to high light stress (Kirst et al., 2014). However, implementations of these strategies rely on antibiotic resistance selection based on genetic manipulations and require long-term cultivations and passages for isolation of the homozygous mutant, and the entire cycle would usually take several weeks.

*Synechococcus elongatus* PCC 7942 (PCC 7942 for short) is a widely used cyanobacterial chassis strain and has been engineered to be cell factories for producing dozens of chemicals, including ethanol (Deng and Coleman, 1999), 2,3-butanediol (Oliver et al., 2013), isobutyraldehyde (Atsumi et al., 2009), ethylene (Sakai et al., 1997), acetone (Chwa et al., 2016), 1-butanol (Lan and Liao, 2011), isopropanol (Kusakabe et al., 2013), 2-methyl-1-butanol (Shen and Liao, 2012), 1,2-propanediol (Li and Liao, 2013), sucrose (Ducat et al., 2012), hexose sugars (Niederholtmeyer et al., 2010), free fatty acid (Ruffing and Jones, 2012), and 3-hydroxypropionic acid (Lan et al., 2015). Moreover, PCC 7942 is a critical platform to explore mechanisms and strategies for optimizing stabilities and efficiency of photosynthetic carbon fixation (Su et al., 2017; Ungerer et al., 2018; Yu et al., 2018). Thus, the improvement of the efficiency and effects on engineering PCC 7942 high light and high temperature tolerances will be of broad and important biotechnological significance.

Previously, we have demonstrated that specific point mutations in F_*O*_F_1_ ATP synthase α subunit (AtpA), converting the 252^*nd*^ amino acid from cysteine to any of the four conjugated amino acids (tyrosine/phenylalanine/tryptophan/histidine), could endow PCC 7942 cells with high temperature and high light resistances (Lou et al., 2018). Inspired by this, we proposed a convenient strategy for rapidly improving high light and high temperature tolerances of PCC 7942 derived cell factories by targeted mutagenesis of AtpA-C252. Compared with previously reported approaches, this new strategy has two potential advantages. First, the direct stress selection (with high light and high temperature) process would eliminate the dependence on introducing and screening antibiotics markers in the recombinant strains. Second, the growth advantages of the desired transformants would reduce the time required for cultivation and segregation. For proof-of-concept, we successfully isolated the PCC 7942 mutant strains with improved high light and high temperature tolerances that could be obtained in only 4 days in an antibiotics-free mode. In addition, cellular robustness and sucrose synthesizing capacities of a previously constructed PCC 7942 cell factory were significantly optimized, increasing the sucrose productivities by nearly onefold.

## Results and Discussion

### Overview

**FIGURE 1:**
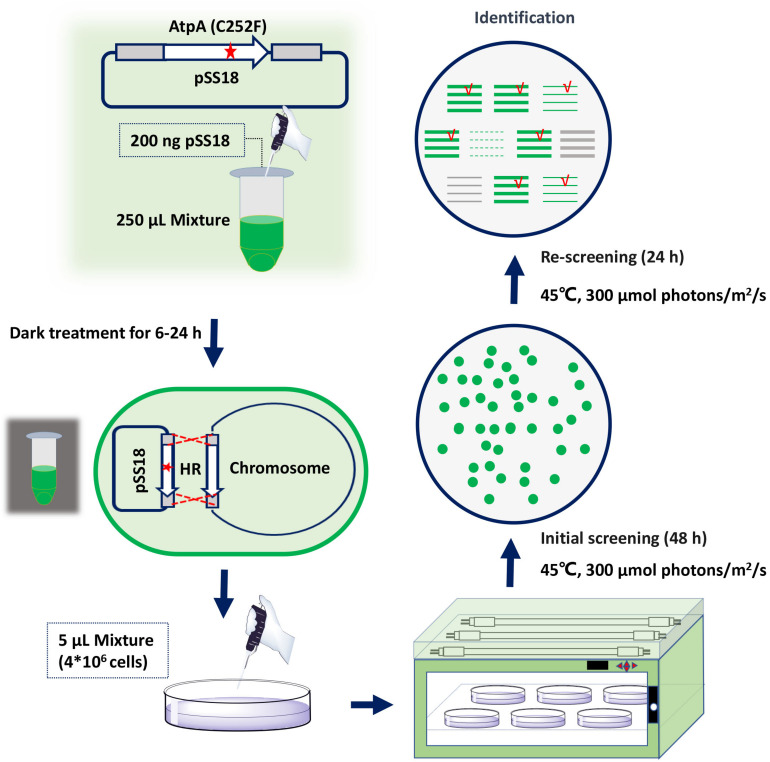
Procedures for rapid isolation of high light and high temperature tolerant PCC 7942 mutants by introduction of the AtpA-C252F mutation.

To facilitate rapid mutagenesis of AtpA-C252 and effective isolation of the mutants, we designed a three-step procedure, consisting of transformation, initial screening, and rescreening. As shown in Figure 1, plasmids carrying tailored AtpA fragments would be transformed into PCC 7942 to induce homologous recombination. During the initial screening step, mutant cells obtaining the AtpA-C252 mutagenesis would survive under the selective conditions and form colonies on the agar plates. To exclude false positive results and to confirm the tolerant phenotypes of the mutants, the colonies obtained from the initial screening step would be streaked and cultivated on fresh agar plates under the same selective conditions. The final colonies would be collected for further assays and evaluations.

### Rapid Isolation of PCC 7942 Mutants Carrying the AtpA-C252F Mutation

Among the four AtpA-C252 mutations endowing PCC 7942 with improved tolerances to high light and high temperature (Lou et al., 2018), we selected AtpA-C252F for proof-of-concept of the proposed strategy in this work. A plasmid carrying an AtpA-C252F gene fragment (termed as pSS18 in Figure 1) was constructed and transformed into PCC 7942 cells, and another plasmid carrying wild-type (WT) AtpA was used as a control. PCC 7942 tolerant transformants appeared on BG11 agar plates after only 48 h of cultivation in the selective conditions with high light and high temperature. More than 1,000 transformants were obtained on the screening plate of PCC 7942 (transformed with pSS18, AtpA-C252F), whereas no transformants appeared on PCC 7942 transformed with pSS3 (AtpA WT). Phenotypes of the transformants were further checked by re-screening under the same conditions, and 23 randomly selected transformants (from the initial screening step) all survived in the rescreening step. We further collected the 23 transformants (after the two-round selection) for AtpA sequence analysis, and the Sanger sequencing results revealed all of the strains carrying the AtpA-C252F mutation, indicating that the tolerances to high light and high temperature were endowed by the targeted mutagenesis. In addition, the sequencing results showed that all the mutants carrying the AtpA-C252F mutation existed as homozygous (as shown in Supplementary Figure 1), indicating that the significant growth advantages caused by the AtpA-C252F facilitated effective segregation of the mutated chromosomes.

**FIGURE 2:**
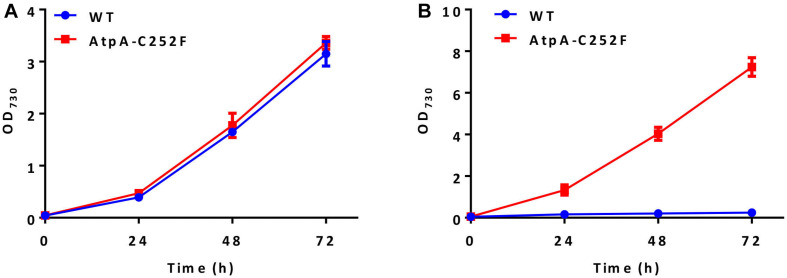
Effect of the AtpA-C252F mutation on cellular growth and tolerances of PCC 7942. Growth of the PCC 7942 strain with or without the AtpA-C252F mutation in liquid BG11 medium at 30°C with 50 μmol photons/m^2^/s illumination **(A)** and at 44°C with 400 μmol photons/m^2^/s illumination **(B)**. Error bars indicate standard deviations (*n* = 3).

High light and high temperature tolerances of the isolated mutants were further evaluated by liquid cultivation in column photobioreactors. As shown in Figure 2A, the growth of the PCC 7942 mutants carrying the AtpA-C252F mutation was similar to that of the control under normal conditions (30°C and 50 μmol photons/m^2^/s). While in a stressful environment (44°C and 400 μmol photons/m^2^/s), the mutant strain exhibited significantly improved adaptabilities compared with the WT control and maintained rapid growth (Figure 2B). WT cells of PCC 7942 could not survive or grow facing the environmental stress, whereas OD_730_ of the AtpA-C252F mutant cells reached up to 7 with a significantly improved growth rate compared with when cultivated in normal conditions (30°C and 50 μmol photons/m^2^/s). The results indicated that the mutant strain of PCC 7942 obtained through the rapid isolation process displayed significantly improved capacities to tolerate and utilize strong illuminations even when cultivated at high temperatures.

### AtpA-C252F Mutagenesis Rapidly Improves High Light and High Temperature Tolerances and Sucrose Production Rates of a PCC 7942 Cell Factory

**FIGURE 3:**
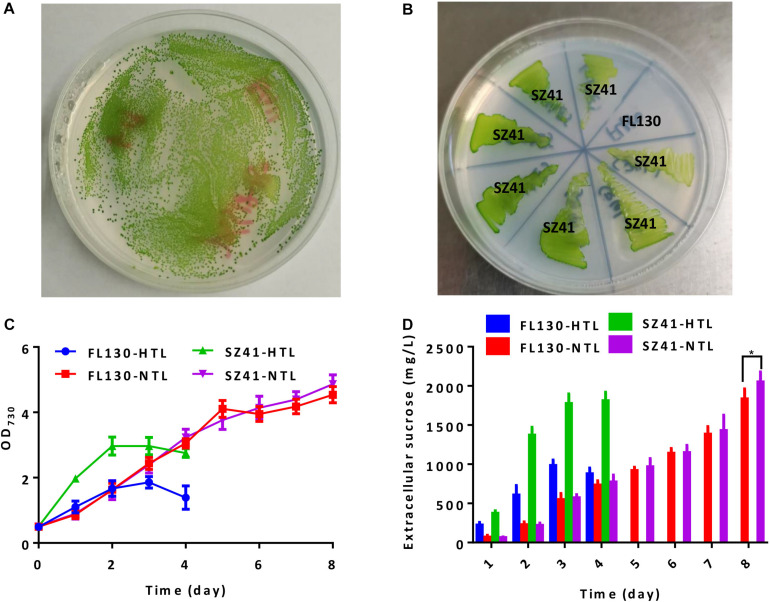
Effects of the AtpA-C252F mutation on growth, tolerance, and sucrose synthesis of a PCC 7942 derived cell factory. **(A)** Tolerant transformants (with the AtpA-C252F mutation) of FL130 on BG11 agar plates after 2 days of cultivation under high light and high temperature conditions (45°C with 300 μmol photons/m^2^/s illumination). **(B)** FL130 and the derived SZ41 strain (FL130-AtpA-C252F) grown on BG11 plates at 45°C with 300 μmol photons/m^2^/s illumination. **(C)** Cell growth of FL130 and SZ41 under normal (NTL, 30°C with 100 μmol photons/m^2^/s illumination) and stressful (HTL, 40°C with 400 μmol photons/m^2^/s illumination) conditions as measured by OD_730_. **(D)** Extracellular sucrose production of FL130 and SZ41. NaCl (150 mM) and IPTG (0.1 mM) were added to the BG11 medium **(C,D)**. Error bars indicate standard deviations (*n* ≥ 3). Statistical analysis was performed by using Student’s *t*-test (*p < 0.05).

We further adopted this strategy to engineer a sucrose synthesizing cyanobacterial cell factory. Previously, we have introduced an *Escherichia coli* sourced sucrose permease CscB into PCC 7942 to facilitate the secretory production of sucrose under salts stress and overexpressed the native sucrose-phosphate synthase Sps to enhance sucrose synthesis (Duan et al., 2016; Qiao et al., 2018). The final strain FL130 was transformed with the pSS18 plasmid (AtpA-C252F) to confirm the feasibility and effectiveness of our strategy when adopted on cell factories. Following the described procedures, we successfully isolated high light and high temperature tolerant transformants of FL130 (Figure 3A). The final isolated and verified FL130 mutant (AtpA-C252F) was termed as SZ41, which was able to grow under the conditions of 45°C with 300 μmol photons/m^2^/s illumination (Figure 3B).

**FIGURE 4:**
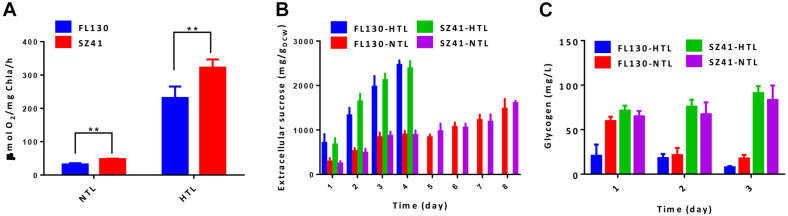
Effects of the AtpA-C252F mutation on carbohydrate production of a PCC 7,942 derived cell factory. **(A)** Oxygen evolution rate of the FL130 and SZ41 strains under normal (NTL, 30°C with 100 μmol photons/m^2^/s illumination) and stressful (HTL, 40°C with 400 μmol photons/m^2^/s illumination) conditions. **(B)** Specific sucrose productivities of the FL130 and SZ41 strains under normal (NTL, 30°C with 100 μmol photons/m^2^/s illumination) and stressful (HTL, 40°C with 400 μmol photons/m^2^/s illumination) conditions. **(C)** Glycogen accumulation of the FL130 and SZ41 strains under normal (NTL, 30°C with 100 μmol photons/m^2^/s illumination) and stressful (HTL, 40°C with 400 μmol photons/m^2^/s illumination) conditions during the initial 3 days of cultivation. Error bars indicate standard deviations (*n* ≥ 3). Statistical analysis was performed by using Student’s *t*-test (**p < 0.01).

When cultivated under normal conditions of 30°C and 100 μmol photons/m^2^/s, growths FL130 and SZ41 show minor differences (Figure 3C, FL130-NTL and SZ41-NTL), and the sucrose production reached up to 1,835 and 2,025 mg/L in 8 days, respectively (Figure 3D, FL130-NTL and SZ41-NTL). The slight increase of total carbon fixation (the sum of biomass and sucrose) in SZ41 might result from an increased oxygen evolution rate (Figure 4A), indicating that the introduction of AtpA-C252F brought in benefits on the overall efficiency or stability of cellular photosynthesis in PCC 7942 under these conditions (30°C, 100 μmol photons/m^2^/s, 150 mM NaCl). When stressful high light and high temperature conditions (40°C and 400 μmol photons/m^2^/s) were adopted, SZ41 exhibited significantly improved growth advantages over FL130. After 4 days of cultivation supplemented with 150 mM NaCl, the cell density of the SZ41 culture broth reached about 3, whereas that of the FL130 was lower than 2.0 (Figure 3C). The bleaching phenotypes might be caused by the synergy effects of high light and high temperature stress and salts stress on the *Synechococcus* cells, and during this process, 1.8 and 0.98 g/L of extracellular sucrose were synthesized by SZ41 and FL130, respectively, under the high light and high temperature conditions (Figure 3D). The different performances on cell growths and sucrose production between the two strains under the stressful conditions are in accordance with the photosynthesis activities differences revealed from the oxygen evolution rates. With enhanced illumination strengths (and increased temperatures), photosynthesis activities of FL130 and SZ41 were both significantly elevated compared with these under normal conditions, and the oxygen evolution rate of SZ41 would be 40% higher than that of FL130 (Figure 4A).

It is noteworthy that the specific sucrose production on per cell levels was on similar levels between the two strains (Figure 4B) whether in normal or stressful conditions, indicating that the improved accumulation of sucrose in the SZ41 strain resulted from the optimized cell growth and enhanced carbon fixation caused by the AtpA-C252F mutation, rather than a distribution of a larger portion of biomass to sucrose synthesis. After 4 days of high light and high temperature cultivation, bleaching phenotypes would be observed for both the SZ41 and FL130 cells, and the sucrose production would be terminated. However, it is noteworthy that the AtpA-C252F mutation carrying the SZ41 strain under the stressful conditions could synthesize similar concentrations of sucrose utilizing half of the cultivation term as that obtained under normal conditions (1.8 g/L in 4 days versus 2.0 g/L in 8 days). In addition to sucrose, the SZ41 strain also accumulated higher intracellular glycogen contents than the FL130 under high light and high temperature conditions, confirming the improved capacities for carbohydrates synthesis caused by the AtpA-C252F mutations (Figure 4C). Both sucrose and glycogen biosyntheses depend on the precursor glucose-1-phosphate, and it has been reported that the metabolic flux through glucose-1-phosphate significantly determines the flexibility of the intracellular carbon distribution of cyanobacteria *via* a dynamic balance between different metabolic branches (Luan et al., 2019). Furthermore, it has been reported that *S. elongatus* UTEX 2973, the fast-growing strain carrying AtpA-C252Y single nucleotide polymorphism (SNP) compared with PCC 7942, would overaccumulate glycogen to buffer the enhanced carbon flux from the CBB cycle (Song et al., 2016; Tan et al., 2018). Glycogen storage serves as the main carbon sink mechanism of cyanobacterial cells and could play the role of carbon pool for synthesis of desired metabolites; thus, the enhanced glycogen synthesis activities and glycogen contents could be an additional advantage of the strategy developed in this work.

We also evaluated the effects of this strategy on engineering an ethanologenic cell factory derived from PCC 7942. A previously optimized ethanologenic pathway consisting of the pyruvate decarboxylase from *Zymomonas mobilis* and a type II alcohol dehydrogenase from *Synechocystis* sp. PCC 6803 was introduced into PCC 7942 to generate the ZN44 strain, producing about 0.22 g/L ethanol in 2 days (Supplementary Figure 2). Introduction of the AtpA-C252F mutation into ZN44 significantly improved the growth of the ethanologenic cell factory (ZN45) under high light conditions (OD_730_ of 7.2 in ZN45 compared with OD_730_ of 5.1 in ZN44 after 2 days of cultivation). However, the ethanol production was just slightly improved from 0.22 to 0.25 g/L, indicating that as for the PCC 7942 derived ethanologenic cell factory, the activities of the ethanol synthesis pathways rather than the total were holding control over the ethanol production capacities, and the increase of carbon fixation could not be effectively rewired into ethanol synthesis, which could be solved through further metabolic engineering.

Compared with the recently reported fast-growing and robust cyanobacteria *S. elongatus* UTEX 2973, PCC 7942 still possesses the advantages of clear genetic background and more convenient genetic manipulation, and dozens of photosynthetic cell factories have been engineered based on this typical model strain. The strategy developed in this work provided a convenient approach to update the related PCC 7942 derived cell factories, which would avoid repeated implementations of the complex metabolic engineering with a new chassis. In addition, the success in this work provided a novel targeted mutagenesis and selective isolation manipulation paradigm for cyanobacterial phenotypes improvements. In recent years, many SNPs have been identified to be related with cellular growth or survival benefits, e.g., acid tolerances (Uchiyama et al., 2015), alcohol tolerances (Hirokawa et al., 2018), high light and high temperature tolerances (Lou et al., 2018), and growth rates (Ungerer et al., 2018). Regarding such mutations endowing cells with specific growth advantages, rapid and convenient transplantation to photosynthetic cell factories could be expected through similar procedures developed in this manuscript, aiming to optimize the growth or tolerance properties. In addition, to some gene deficiency-related growth advantages (meaning benefits caused by loss of specific gene functions) (Joseph et al., 2014; Kirst et al., 2014), this strategy could be expected to work and facilitate the rapid isolation of the gene-deficient mutants. However, it is still noteworthy that the application of this strategy to other targets might be limited by the significant degrees of the growth advantages endowed by specific mutations, which might not be as effective as that from AtpA-C252 (stress tolerances coupled with growth advantages). To this end, systematic optimization of selective pressure strengths and methods would be required in order to achieve the ideal effects of strain improvements.

## Conclusion

High light and high temperature tolerances are important properties for cyanobacterial cell factories, aiming to put the photosynthetic biomanufacturing technology into practice. We developed a rapid and convenient strategy for improving cellular tolerances to high light and high temperature in an important cyanobacterial chassis PCC 7942 derived cell factory by antibiotics-free introduction of an AtpA-C252F mutation. Adopting this strategy, cellular robustness and sucrose synthesizing capacities of the PCC 7942 derived cell factory were significantly improved.

## Materials and Methods

### Chemicals and Reagents

All reagents were purchased from Sigma-Aldrich (United States). *Taq* and *pfu* DNA polymerases for PCR were purchased from Transgene Biotech (Beijing, China). T4 DNA ligase and restriction enzymes were purchased from Fermentas (Canada) or New England Biolabs (Japan). The kits for molecular cloning were from Omega Bio-tek (Norcross, GA, United States). Oligonucleotides were synthesized, and DNA sequencing was performed by Tsingke (Qingdao, China).

### Construction of Plasmids and Strains

*Escherichia coli* DH5α (TaKaRa, Dalian, China) was used as a host for cloning. The strains constructed and used in the present study are listed in Table 1. All the constructed plasmids employed pUC19 as backbone. To construct pSS3 and pSS18, AtpA-encoding gene containing 252C/252F was cloned from the chromosome of PCC 7942 and PCC 7942-C252F *via* PCR using the primers AtpA-UP500-F-*Nde*I and AtpA-Down500-R-*Eco*RI listed in Table 2. PCR products were purified using the E.Z.N.A Cycle Pure Kit (Omega Bio-Tek, Norcross, GA, United States), and then the AtpA/AtpA-C252F fragments were digested with *Nde*I and *Eco*RI and inserted into pUC19 for constructing the target plasmids pSS3 and pSS18. To construct the ZN44 strain, a cassette containing the spectinomycin resistance gene, the *PrbcL* promoter sequence, the *Pdc*_*ZM*_ gene, and the *slr1192* gene was amplified from a previously constructed pZG25 plasmid (Gao et al., 2012) and ligated with the backbone of another previously constructed plasmid pFL20n (Duan et al., 2016) containing the upstream and downstream sequences of neutral site I (NS1). The final obtained plasmid was termed as pZN44. All the resulting plasmids were then verified by Sanger gene sequencing and are listed in Table 1.

**TABLE 1:** Cyanobacterial strains and plasmids used in this study.

Strain	Genotypes^*a*^	References
PCC 7942	*Synechococcus elongatus* PCC 7942 wild-type	Gifted by Prof. Xudong Xu, Institute of Hydrobiology, Chinese Academy of Sciences
PCC 7942-C252F	Mutant strain of PCC 7942 harboring a AtpA with C252F point mutation	Lou et al., 2018
FL130	Mutant strain of PCC 7942 harboring NS1::*P*_*trc*_-*sps* and NS3::*P*_*trc*_-*cscB*, Sp^*r*^ Cm^*r*^	Duan et al., 2016
SZ41	Mutant strain derived from FL130, carrying atpA with the C252F point mutation	This study
ZN44	Mutant strain of PCC 7942 harboring NS1::*P_*rbcL*_-pdc_*ZM*_-slr1192*, Sp^*r*^	This study
ZN45	Mutant strain derived from ZN44, carrying AtpA with the C252F point mutation	This study
Plasmids		
pSS3	Carrying the wild-type PCC 7942 *atpA* gene	This study
pSS18	Carrying the PCC 7942 *atpA* mutant gene with the C252F mutation	This study
pZN45	Carrying the NS1UP-Sp^*r*^-*P_*rbcL*_-pdc_*ZM*_-slr1192*-NS1DOWN cassette	This study

*^*a*^*P*_*trc*_, *trc* promoter; *cscB*, proton/sucrose exporter gene from *Escherichia coli*; *sps*, encoding a natively fused protein of sucrose-phosphate synthase SPS; NS1 and NS3, different neutral sites in the genome of PCC 7942; Ap^*r*^, ampicillin resistance; Cm^*r*^, chloramphenicol resistance; Sp^*r*^, spectinomycin resistance.*

**TABLE 2:** Primers used in this study.

Primer	Sequence (5′→3′)	Purpose
AtpA-UP500-F-*Nde*I	AAATTCATATGGGATGCAGCCCTATTCCGAA	Amplifying the *atpA* gene
AtpA-Down500-R-*Eco*RI	AACCTGAATTCCGGGATTTGCTCCAAACCAC	Amplifying the *atpA* gene
AtpA-before-F	GATTTCGATCAGTTTGGCCGCC	Checking AtpA genotypes of *Synechococcus elongatus* PCC 7942 mutants
AtpA-after-R	TCGCGAATCGCCTTGAGGTTTG	Checking AtpA genotypes of *Synechococcus elongatus* PCC 7942 mutants
pSN44-NS1-r	TTTGTTCGCCCAGCTTCTGTATGGCTCGAGCTTCT GGAGCAGGAAGATGT	Amplifying the backbone of pSN44
pSN44-NS1-f	TGCTCAGCCATAGTAAAAATTAGTCCCTGCTCGTC ACGCTTTCAGGCACC	Amplifying the backbone of pSN44
pSN44-slr1192-r	CTAATTTTTACTATGGCTGA	Amplifying the Sp^*r*^-*P_*rbcL*_-pdc_*ZM*_-slr1192* DNA fragment
pSN44-sp-f	GCATGCCCGTTCCATACAGA	Amplifying the Sp^*r*^-*P_*rbcl*_-pdc_*ZM*_-slr1192* DNA fragment

### Transformation of the PCC 7942 Strains

Transformation of PCC 7942 derived cell factory FL130 was performed according to a previously reported method with optimization and quantitation (Lou et al., 2018). Briefly, 2 ml WT or FL130 broth with an OD_730_ of 1.0 was centrifuged at 6,000 × *g* for 5 min and then re-suspended in 250 μl of fresh BG11 medium. Next, 200 ng plasmid was added and incubated in the dark at 150 rpm and 30°C for 6–24 h. Then, 5 μl of the mixtures (about 4 × 10^6^ cells) was plated on solid BG11 plates with 1.5% agar after transformation and incubated at 45°C and 300 μmol photons/m^2^/s for 2 days. The obtained colonies would be picked, streaked on fresh BG11 plates, and incubated for another 24 h under the same conditions. Genotypes of the final survived transformants would be checked, and the *atpA* gene would be amplified for Sanger sequencing.

### Cultivations of Cyanobacterial Strains

For phenotypes evaluations, PCC 7942 WT and the derived strains would be cultivated in BG11 medium with column photobioreactors (3 cm diameter), and the temperature and illumination strengths would be set as required. The light would be provided by incandescent lamps (NVC, NDL433SI-28W). Then, 3% (volume to volume) CO_2_ air would be bubbled for carbon source supplementations. For liquid cultivation of the WT PCC 7942 and the derived mutant carrying the AtpA-C252F mutation, the normal conditions were set as 30°C and 50 μmol photons/m^2^/s, and the respective stressful conditions were set as 44°C and 400 μmol photons/m^2^/s. For sucrose production, 150 mM NaCl and 0.1 mM isopropyl-D-1-thiogalactopyranoside (IPTG) would be added to the culture medium. During this process, normal conditions were set as 30°C and 100 μmol photons/m^2^/s, whereas stressful conditions were set as 40°C and 400 μmol photons/m^2^/s. The growth of each cyanobacterial strain was monitored by measuring the optical density at 730 nm (OD_730_). Dry cell weights (DCWs) were determined following the methods previously described (Wang et al., 2019). As for the cultivation of ethanologenic strains, the conditions were also set as 30°C and 400 μmol photons/m^2^/s, and the ethanol production would be evaluated as previously described (Wang et al., 2019).

### Oxygen Evolution Rates Determination

Oxygen concentration was measured using fiber-based and contactless oxygen microsensors (PyroScience, Aachen, Germany). The sensor was calibrated with air-saturated water and de-oxygenated water as 100% and 0% O_2_ levels. The culture broths of FL130 or SZ41 were transferred to the respiration vials and placed in the corresponding culture conditions. The oxygen concentration in the respiration vials was continuously monitored by the oxygen microsensor connected to the oxygen logger software. The final photosynthetic oxygen evolution rate was calculated according to the *Chl*a content, which was determined spectrophotometrically at OD_665_ and OD_720_ in methanol extracts and calculated with the formula: *Chl*a (mg/L) = 12.9447 × (A665 − A720).

### Determination of Sucrose and Glycogen Contents in PCC 7942

To determine the amount of extracellular sucrose in PCC 7942 culture broth, 1 ml of cyanobacterial culture was centrifuged, and sucrose in the supernatant was measured using the sucrose/D-glucose assay kit (Megazyme). The glycogen contents in PCC 7942 cells were measured as previously introduced with modifications (Chi et al., 2019). After the glycogen precipitants were hydrolyzed to glucose by treatment with amyloglucosidase at 60°C for 2 h, glucose in 100 mM sodium acetate (pH 4.5) solution was measured using the sucrose/D-glucose assay kit (Megazyme).

## Data Availability Statement

The raw data supporting the conclusions of this article will be made available by the authors, without undue reservation.

## Author Contributions

SZha, SZhe, XZ, and YD performed the research project. GL and XL supervised the research project and guided the design of experiments. SZha, GL, and XL drafted and revised the manuscript. All authors contributed to the article and approved the submitted version.

## Conflict of Interest

The authors declare that the research was conducted in the absence of any commercial or financial relationships that could be construed as a potential conflict of interest.
