# Supplementary material for: Rapidly Improving High Light and High Temperature Tolerances of Cyanobacterial Cell Factories Through the Convenient Introduction of an AtpA-C252F Mutation
Source: Front Microbiol. 2021 Apr 8;12:647164. doi: 10.3389/fmicb.2021.647164 (PMC8060558; doi:10.3389/fmicb.2021.647164)
Supplement: Supplementary file 1 [file Data_Sheet_1.docx]

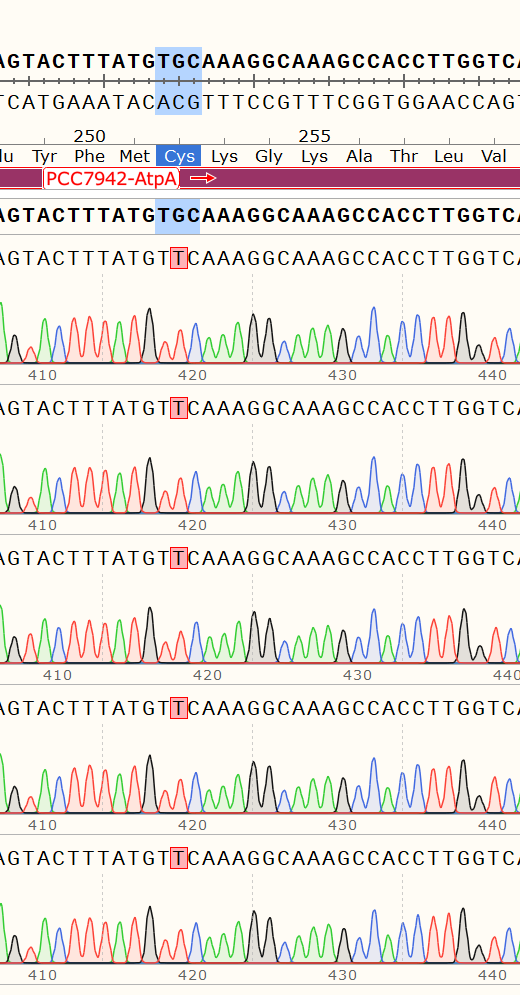


**Figure S1.** Sanger DNA sequencing of the amplified AtpA region of 5 randomly isolated AtpA-C252F mutants.


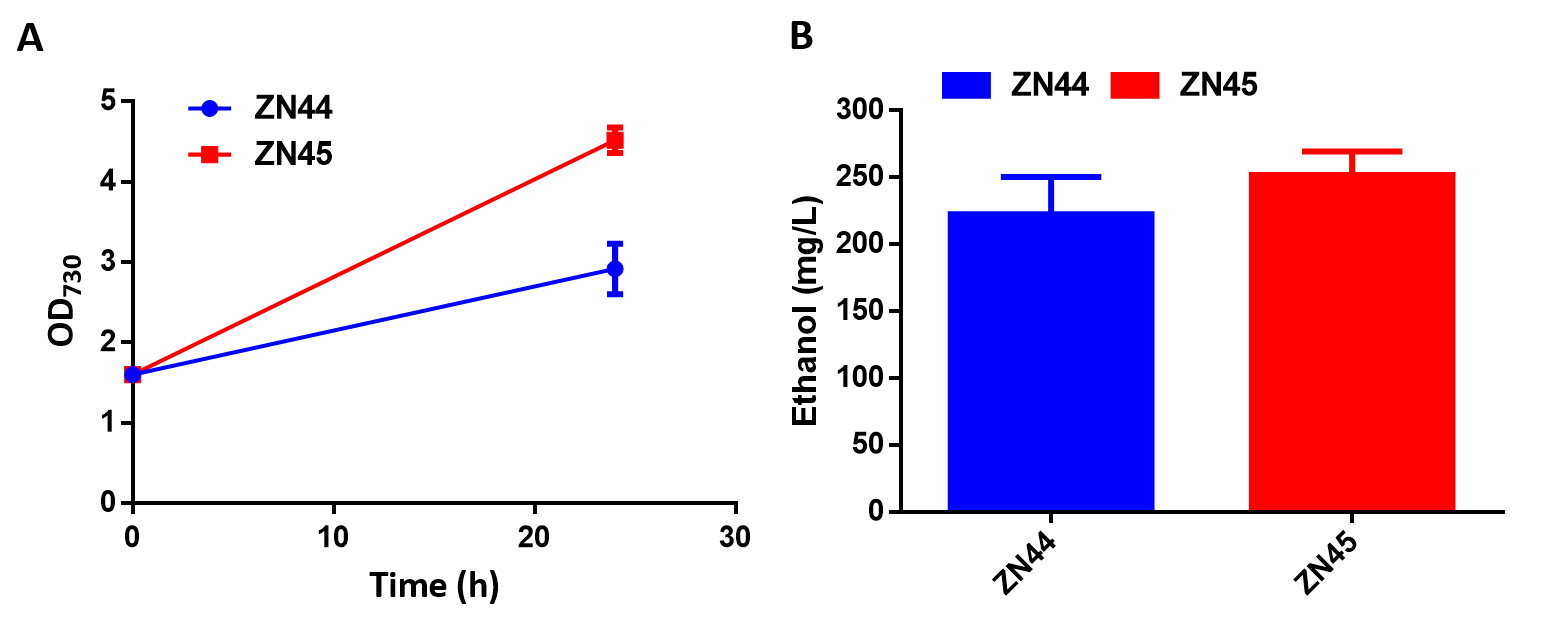
Figure S2. Effects of the AtpA-C252F mutation on growth and ethanol synthesis of a PCC 7942 derived cell factory. (A) Cell growth of ZN44 (PCC 7942 carrying a *PrbcL-Pdc_ZM_-slr1192* cassette on NSI) and ZN45 (ZN45 carrying an AtpA-C252F mutation) under stressful (30^o^C with 400 µmol photons/m^2^/s illumination) condition as measured by OD_730_. (B) Ethanol production of ZN44 and ZN45. Error bars indicate standard deviations (n≥3).
